# Women’s intuition as attunement: a biological and social perspective

**DOI:** 10.3389/fsoc.2025.1542563

**Published:** 2025-09-23

**Authors:** Shalini Verma, Lucy (Raiya) Kind

**Affiliations:** Google, Mountain View, CA, United States

**Keywords:** intuition, attunement, evolution, women, embodied experience, embodied cognition, healing, trauma

## Abstract

This paper proposes that women, due to both evolutionary pressures and sociocultural reinforcement, have developed heightened intuitive capacities, more specifically, attunement capabilities. We define attunement as the ability to somatically, emotionally, and cognitively resonate with the internal states of oneself or another. It is especially important in contexts involving caregiving, safety detection, and emotion regulation. Crucially, this ability is not static; it can be dulled or distorted by traumatic experience and reclaimed through healing. Through the restoration of nervous system balance, individuals, and especially trauma survivors, may gain more accurate and deeper information and thus greater access to their intuition. By grounding female intuition in the body and relational experience, we position it as a vital, learnable, and reparative human skill, particularly important in—but not limited to—caregiving, therapy, decision making, and community life. Finally, we stress that since attunement is an ability innate to all humans and not just women, men too can heighten their attunement abilities, and thus their intuition, and we as a society can encourage all beings to increase this potent and beneficial ability.

## Introduction

Intuition is often described as immediate knowing without conscious reasoning (Epstein, 2010). This paper focuses on a specific form of intuition: *attunement*, defined here as the ability to somatically, emotionally, and cognitively resonate with the internal states of oneself or another (Stern, 1985; Hübl, 2023). Attunement is a biologically grounded and relationally cultivated capacity, rooted in the nervous system’s ability to track arousal, interpret subtle cues, and co-regulate with others (Siegel, 2019; Hübl, 2023). In some situations, such as the mother–child relationship, attunement functions as the primary form of intuition (Feldman et al., 2011), whereas in others, attunement is a component of intuition (Decety and Jackson, 2004). Our hypothesis is that through mechanisms of nature (biology) and nurture (cultural reinforcement), women have developed higher intuitive abilities, and specifically attunement abilities, compared to men. We propose that this heightened intuitive capacity results from a complex interplay of biological and sociocultural factors.

Biologically, women bear the primary responsibility for carrying a child to term. We posit that those individuals with heightened sensitivity to their infants were better able to recognize subtle signs of sickness in order to learn to avoid foods and other environmental situations that are harmful, as well as recognize conditions that enhance well-being. As they integrated these cues and their associated meanings into subconscious knowledge, they increased the likelihood of survival for both themselves and their offspring (Nordin et al., 2004). Over generations, it was recognized that this inner sensitivity was advantageous in relation to female-specific tasks such as child rearing and social bonding, and thus heightened intuition was a virtue that was both sought after and encouraged in women. Additionally, female-specific experiences like pregnancy (Hoekzema et al., 2017) and menstrual cycles (Pritschet et al., 2020; Andreano and Cahill, 2009; Childs et al., 2010) necessitate a deeper inward focus, fostering heightened sensitivity and attention to bodily sensations (Pearson et al., 2009), often called interoception (Craig, 2002).

Culturally, modern Western society tends to be more accepting and encouraging of emotional expression in women (Fischer and LaFrance, 2015), which facilitates a connection with their bodies, further enhancing sensitivity (Khalsa et al., 2009; Ainley et al., 2013; Gendlin, 1978). Women are also more likely to engage in therapy (Addis and Mahalik, 2003), where they may recognize past trauma—i.e. “unprocessed memories from distressing event(s) that can cause lasting harm to mental, physical, and emotional well-being” and negatively affect their present day experience—which they can address and heal (van der Kolk, 2014). Since trauma can dull sensitivity as a protective mechanism (or, alternatively, greatly distort it), the process of healing allows women to reclaim and even heighten their somatic awareness, further boosting their intuitive capabilities.

We posit that women, through a confluence of biologically-specific roles, hormonal influences, and cultural expectations, have developed heightened intuitive attunement capacities. We explore how this capacity emerges from ancestral experiences such as pregnancy, childbirth, and maternal bonding, and is then reinforced through sociocultural conditioning, emotional socialization, and trauma healing. Furthermore, we suggest that while trauma can sever this capacity, healing can restore and even refine it, allowing individuals to reinhabit their intuitive intelligence with greater sensitivity and resilience.

Drawing from neuroscience, attachment theory, trauma studies and embodied cognition, this paper reframes intuition as a relational skill: one that is biologically scaffolded, socially rehearsed, and emotionally reparable. In so doing, we position intuition not as a mere soft skill, but as a vital survival strategy that is essential for human thriving. Finally, just as attunement and the enhanced intuitive receptivity it engenders (Isenman and Sinclair, 2025) has been encouraged and honed by women across generations, we propose that men can also increase their development of this innately human ability, and thereby benefit from the life-supporting capacity of this ancient wisdom.

We will explore these factors of nature and nurture, and in particular how they relate to embodiment and attunement, in the sections below.

## Theoretical framework

### Biological gender vs. gender identity

This paper includes a holistic exploration of biological differences between male and female embodied experiences. We use the term “women” to refer to biological females (e.g., beings that have the biological equipment for childbirth) and “men” to refer to biological males. We also recognize that those who choose their own gender identity might not subscribe to these terms, and we use them solely for the sake of clear language to discuss these unique embodied experiences within the confines of this paper.

### The intuition of attunement

We focus specifically on attunement, which is a form of intuition grounded in somatic-emotional-cognitive resonance, frequently involving nervous system synchronization with another. Throughout the paper, when we use the word “intuition” we do so primarily in reference to the specific type of intuition known as “attunement.”

Attunement builds on the foundation of early caregiver-infant bonding, as described in attachment theory (Bowlby, 1988; Ainsworth et al., 2014), where an infant’s unregulated nervous system is stabilized through maternal presence. These early attunement experiences create embodied patterns of emotional regulation and relational perception that endure into adulthood (Schore, 2003). Recent imaging studies support this biologically-rooted form of communication; for example, Nguyen et al. (2020) demonstrate neural synchrony between mothers and infants during face-to-face interaction, while Kinreich et al. (2017) showed similar synchrony between adults in emotionally resonant exchanges. Such findings suggest that nervous system attunement remains a vital mechanism for social connection and understanding across a person’s lifespan.

These and other recent studies in interpersonal neuroscience offer compelling evidence for the biological basis of attunement as a real-time, embodied phenomenon. Far from being metaphorical, attunement can now be measured as brain-to-brain synchrony—a dynamic alignment of neural activity between individuals during social interaction particularly in the temporo-parietal junction (TPJ), a brain region associated with empathy and theory of mind (Kinreich et al., 2017).

### The integration of embodied experience

Our framework incorporates the notion of embodied cognition, which posits that the body, brain, and environment collectively shape cognitive processes (Shapiro and Spaulding, 2021). However, we expand upon this notion by emphasizing *embodied experience*, a more visceral and intuitive process that reflects lived, bodily engagement with the environment and with others. While “embodied cognition” traditionally refers to how the body contributes to thought processes the emphasis on “embodied experience” (Varela et al., 1991; Gallagher, 2005) highlights the direct felt sense and grounds attunement in moment-to-moment bodily awareness. By focusing on intuition as attunement (Gendlin, 1978; Isenman, 2018, 2020), we argue it is the result of nervous system sensing and responsiveness: the subtle tightening of one’s gut, the flush of the skin, the quickened breath when sitting with another’s distress. Sensory and interoceptive cues, e.g., heartbeat, breath, muscle tension, internal temperature, form the data streams that help inform attunement (Garfinkel et al., 2015).

We argue that women’s heightened sensitivity and intuitive capacities represent a form of somatic (bodily) intelligence that evolved in response to the demands of their childrearing and caregiving roles, i.e., from their embodied experience as women. More specifically, we posit that the adaptive processing of emotions has a key role in generating the embodied knowledge that informs intuition, and especially women’s intuition. William James’s theory holds that emotions are the mind’s perception of physiological changes in response to stimuli related to survival (James, 1884). Furthermore, Damasio (1994) argued that personal decision making is to a large extent dependent on emotional memories, which, as he stresses, are body-tinged memories based on what has proved disadvantageous or advantageous in the past. Thus, bodily sensations can provide meaningful information about the external world, including other people (Quadt et al., 2018), which can help guide intuition. Think of the body in part as an instrument for receiving and processing important information (visual data, chemical data, audio, etc.) about our surroundings and translating this data into our internal knowledge (emotional and other visceral sensations along with their meanings).

Much of this embodied knowledge is implicit (Cleeremans and Jiménez, 2002) and helps instruct our understanding from below awareness (Isenman, 2018). It can strongly influence attunement without coming into consciousness, although if it does surface into awareness, its potential impact may be greatly magnified. Furthermore, the more we attend consciously to bodily sensations and their meaning, the more we also encourage our intuition, which integrates largely below awareness, to ground all its understanding in embodied knowledge (Gendlin, 1978; Isenman, 2020), greatly enhancing its richness and resonance. From this perspective, intuition, and thus attunement, become a form of embodied knowledge that integrates physiological, emotional, and cognitive signals in real time.

## Biological foundations of women’s intuition

Attachment theory provides a compelling biological framework for understanding the roots of attunement. Bowlby (1969, 1988)proposed that human infants possess an innate attachment system that motivates proximity to caregivers when under stress. Furthermore, Ainsworth et al. (2014) demonstrated how a caregiver’s ability to attune to the infant’s emotional and physiological signals determines the quality of the attachment bond—secure, avoidant, or anxious. Secure attachment, rooted in consistent somatic co-regulation, leads to healthier emotion regulation, reduced physiological reactivity to stress, and greater empathic capacity in adulthood (Schore, 2003; Siegel, 2012). These early experiences become biologically embedded, influencing the development of the prefrontal cortex, limbic system, and autonomic nervous system which all support intuitive sensing and relational perception (Schore, 2003; Siegel, 2012).

We examine the fundamental biological differences between men and women that led to distinct roles in early human societies. Men, with generally larger and stronger bodies that correlate to increased knockout power, were biologically suited for fighting and protection (Caton et al., 2024). Women, with capacity for childbearing and nursing, were naturally equipped for caregiving roles (Hallers-Haalboom et al., 2017). We propose that women’s intuition developed as an adaptive trait specifically suited to the demands of child bearing and rearing, and more generally of caregiving. Women possessing increased intuition were likely more successful in nurturing and protecting their offspring, leading to better outcomes for their children.

In women, attunement has become a refined and vital form of intuition, demanded by their biological and cultural experience. Attunement is biologically rooted in the human capacity for nervous system-to-nervous system synchrony, a relational process that emerges in early development and continues throughout the lifespan (Feldman, 2012). From birth, an infant’s survival depends on nonverbal communication through bodily signals, i.e., shifts in muscle tone, cries, breath rhythms, skin temperature, and scent which are sensed and responded to through the mother’s own nervous system, forming a feedback loop of co-regulation and intuitive understanding. This somatic synchrony, sometimes called bio-behavioral synchrony, is observable in heart rate variability, hormonal responses, and neural activation patterns (Feldman, 2012; Nguyen et al., 2020) during moments of close connection. Recent research confirms that synchrony continues into adulthood in varied forms whether through shared emotional experiences, joint action, or shared meaning (Dumas et al., 2010; Hasson et al., 2012).

Over the course of human evolution, women who could more finely detect and respond to these bodily cues were more likely to ensure the survival of their offspring by being able to better attend to the real-time needs of the infant. Olfaction plays a particularly crucial role in this process: Mothers can detect subtle changes in their infant’s scent—often signaling illness, hormonal shifts, or emotional distress—before any overt behavior appears. This sensitivity to smell, along with touch, vocal tone, and movement, became adaptive traits for early detection and intuitive caregiving. These capacities were then reinforced through selective pressure. Notably, olfactory signals arrive at the limbic system more directly than other sensory signals (Kandel et al., 2000), a brain region typically associated with memory and emotional processes, giving scent a uniquely direct influence on intuitive awareness (Sullivan et al., 2015).

Neuroscientific research also shows that women, on average, display greater activity in brain regions associated with interoception, empathy, and emotional regulation—particularly the insula, anterior cingulate cortex, and amygdala (Young and Barrett, 2015). These structures allow for real-time monitoring of both internal bodily states and subtle shifts in others, enhancing relational attunement. Studies have shown that women possess superior accuracy in detecting emotional expressions, especially those signaling potential danger; for example, Pearson et al. (2009) demonstrated that this increased emotional sensitivity is particularly evident during late pregnancy and early motherhood, suggesting it plays a crucial role in protecting and nurturing offspring. In addition, longitudinal studies by Hallers-Haalboom et al. (2017) indicate that mothers consistently exhibit higher sensitivity in interactions with their children compared to fathers, which implies greater maternal attunement when caring for their children’s specific needs. This finding lends further support to the evolutionary roots of women’s intuitive development in relation to caregiving, where women’s heightened emotional sensitivity leads to intuitive benefits to ensure survival of offspring.

In addition to evolutionary shaping, women undergo recurring physiological cycles that deepen their capacity for embodied awareness. Biologically, females alone go through a monthly cyclical experience of menstruation; such cyclical fluctuation of physical, emotional, and mental experience would therefore bring women’s attention to their shifting internal state. For example, Pritschet et al. (2020) found that different phases of the menstrual cycle correspond with structural changes in brain tissue linked to cyclically fluctuating hormonal levels like estradiol and progesterone; Both estradiol (associated with enhanced emotional recall) and progesterone (linked to increased stress sensitivity) can amplify self-awareness and emotional perception (Andreano and Cahill, 2009; Childs et al., 2010). Given that these brain and bodily alterations are cyclical and therefore predictable, we argue that by experiencing these repeating cycles, women gain awareness of such internally changing states, much like how someone who lives in a place with extreme seasons becomes attuned to annual temperature and humidity shifts. Over time, such embodied experiences and resulting self-attunement may scaffold broader interpersonal attunement (Pritschet et al., 2020; Andreano and Cahill, 2009; Childs et al., 2010).

These embodied capacities are especially intensified during late pregnancy and early motherhood. Hormonal changes, including increased levels of oxytocin activate neural networks that heighten emotional sensitivity, bonding, and vigilance (Leuner et al., 2011). Brain imaging studies show increased activation in the amygdala, insula, and prefrontal cortex during this period, priming mothers to detect minute affective cues and nonverbal signals in their infants (Kim et al., 2018). These neurobiological shifts support not just caregiving behaviors, but deep attunement on a nervous system level.

Biological sex hormones also play a role in modulating attunement capacities. Testosterone, typically higher in men, has been linked to reduced empathic accuracy and decreased sensitivity to emotional expressions (Hermans et al., 2007; van Honk et al., 2011). Elevated testosterone levels are associated with lower activity in brain areas involved in interoception and emotional resonance, such as the insula and inferior frontal gyrus (van Honk et al., 2011). These findings underscore how hormonal profiles can affect intuitive responsiveness across different life stages and contexts, and also highlight that women’s attunement abilities tend to be better preserved than that of their male counterparts.

While the capacity for attunement exists across all genders (Chatel-Goldman et al., 2014; Zeevi et al., 2022), including male–female and male–male pairs (Cohen et al., 2024; Pfaus et al., 2023), its expression is profoundly shaped by biological and developmental factors that have allowed for a heightened development of attunement in women. The above evidence illuminates that the roots of intuitive attunement lie in the nervous system itself—scaffolded by evolution, refined through sensory channels like smell and touch, and amplified through the hormonal orchestration of the female life cycle.

In the next section, we will explore how women’s attunement has been further supported and encouraged by cultural practices that promote emotional expressiveness and interpersonal care.

## Sociocultural reinforcement of intuition

While the capacity for intuition is innate to all humans, its expression and level of development is significantly shaped by culture. Building upon the evolutionary and neurobiological roots outlined above, this section explores how cultural norms, gender socialization, and relational roles reinforce and refine intuition among women.

Throughout generations, as women with higher levels of intuition were naturally more successful in birthing and raising surviving offspring, it follows that increased intuition would become a desired trait in a female mate. Thus, society would act to reinforce women’s development of intuition, embodied experience, heightened sensitivity, and emotional awareness. We focus here on examining these traits in western modern society, looking at how gender-specific behaviors are reinforced based on sociocultural context. Sociocultural context refers to the combination of social and cultural factors that influence an individual’s behavior, attitudes, and perceptions including customs, beliefs, values, norms, beliefs and social structures related to specific life events like pregnancy and childbirth. Below, we will examine how gendered sociocultural norms reinforce behaviors in women that subsequently lead to increased intuition.

In many societies, expectations of heightened attunement are woven into gendered roles, where empathy and nurturance are more commonly emphasized for women, while men are often taught to suppress emotional displays (Fischer and LaFrance, 2015). For instance, studies show that from an early age, girls are more likely to be encouraged to express emotions, recognize others’ feelings, and adopt caretaking behaviors; this emotional socialization is embedded in daily interactions, toys, media, and family dynamics, creating a developmental pathway where empathy and relational sensitivity are not only valued but expected (Brody and Hall, 2008; Fischer and LaFrance, 2015). Over time, these early practices train the nervous system to track subtle cues in others, aligning with the mechanisms of attunement described in neurobiological research. Furthermore, as we noted before, studies show that women tend to exhibit greater accuracy in reading nonverbal emotional expressions and are more responsive to relational cues (Hoffmann et al., 2010). This is not solely due to innate ability, but it is also the result of consistent social reinforcement. In mixed-gender interactions, for example, women often modulate tone, manage social harmony, and adapt behavior based on others’ needs. Thus we see that the cultivation of intuition in women is not just encouraged but often valued as a key component of effective caregiving and social interaction, especially in roles involving family and community (Miller, 1986). This repeated exposure to emotionally charged situations especially in caregiving, education, and service-oriented roles lead to interpersonal attunement. Intuition in this view is a refined skill, developed through daily practice and relational feedback. These findings mirror the neuroscience of interpersonal synchrony (Kinreich et al., 2017; Dumas et al., 2010; Hasson et al., 2012), suggesting that culture does not merely reinforce behavior but actively shapes the neural and physiological systems underlying intuition.

For instance, Brody and Hall (2000) explain that women are often encouraged to develop empathic accuracy and emotional expressiveness as part of their gender role, leading to an enhanced awareness of others’ emotional states and the social environment. This can also lead women to an increased awareness of their own bodily experience and emotional states. Furthermore, work by Gendlin (1978) emphasizes that enhancing awareness of bodily sensations can deepen emotional and cognitive understanding, enhancing intuitive capabilities. Such research aligns with the idea that sensory and emotional awareness, influenced by hormonal and social factors, can contribute to more refined intuitive processes (Quadt et al., 2018).

Studies have shown that women tend to outperform men in recognizing emotions from facial expressions, particularly when the expressions are subtle or low in intensity (Hoffmann et al., 2010). This ability, which plays a critical role in social cognition, helps account for what is often perceived as heightened interpersonal intuition in women. These repeated experiences deepen intuitive capacities by fostering embodied pattern recognition: Over time, women learn to anticipate emotional shifts before they are explicitly communicated.

Women’s heightened attunement also offers them the opportunity to identify their own emotional-behavioral response patterns. Since women are more encouraged to be in touch with their inner emotional states, they are more likely to seek therapy than men (Addis and Mahalik, 2003), with data from the United States National Center for Health Statistics in 2019 showing that approximately 24.7% of US women received mental health treatment in the past 12 months, compared to 13.4% of men (Terlizzi and Zablotsky, 2020). This difference based on gender is significant given that therapy has the capacity to significantly enhance sensitivity and intuition (Siegel, 2001). Women are also considerably more likely than men to engage in embodied practices such as yoga and meditation, which can enhance interoceptive awareness and bodily sensitivity (Cramer et al., 2018). These modalities foster body–mind integration and emotional processing, both key to restoring or enhancing intuitive capacity. In particular, therapeutic spaces often serve as relational laboratories where attunement is explicitly modeled, repaired, and internalized through co-regulation with a therapist (Ogden et al., 2006; Siegel, 2012). Thus, we see how a society that encourages women to engage in embodiment-increasing practices allows them to further integrate their embodied experiences and thereby increase their intuitive capacity.

It becomes apparent that women’s heightened intuition is also the result of repeated social, emotional, and physiological practice. Intuition, then, is not a fixed trait but a learnable, context-sensitive capacity, one that is fostered by embodied relational experience, reinforced through socialization, and developmentally encouraged through practices like therapy, yoga, and meditation that integrate embodied experiences into a more attuned nervous system.

## Connection to intuition through healing

Since women have heightened attunement to emotion and sensation, they also may have heightened emotional reactions. These heightened reactions may create or reinforce trauma–repeating habit patterns or maladaptive neural pathways–which If not properly processed, may lead to PTSD (Ehring and Quack, 2010). Indeed, women have nearly twice the lifetime prevalence of PTSD compared to men, despite men experiencing more physical violence (Olff, 2017). Since trauma impairs emotional and sensory processing, which in turn distorts embodied experience, it follows that trauma stored in the body can inhibit intuition. For example, van der Kolk (2014) research on trauma’s impact on the body demonstrates how traumatic experiences, by impairing emotional and sensory processing and limiting one’s access to internal cues, can lead to dissociation from the body, and hinder intuitive capacities (van der Kolk, 2006, 2014).

Trauma disrupts the body’s capacity to signal clearly. It fragments awareness, often leading to dissociation, hypervigilance, or emotional numbing—states that sever the connection between sensation and meaning, between the body’s intuitive cues and the self’s ability to recognize them (Harricharan et al., 2021). When this internal feedback system is impaired, the body is no longer a reliable instrument for sensing safety, connection, or emotional truth. Yet therapy that addresses these disruptions with interventions, such as somatic experiencing or Eye Movement Desensitization and Reprocessing (EMDR), is able to restore interoceptive awareness and improve social cognition and decision-making (Schore, 2003; Levine, 1997). Such embodied modalities enhance the ability to sense and interpret many subtle signals, for example, subtle social signals, thus reinforcing intuitive abilities. Indeed, Ogden et al. (2006) show that healing from trauma restores emotional health and re-establishes connection with internal cues and sensations, essential for intuition. Thus, given that women are twice as likely to seek therapy than men, it follows that more women would engage in activities that help develop intuitive capacities than men would overall. As we strengthen our mind–body connection and become somatically aware, we reconnect to the internal signals and data that are integral for attunement and intuition. Healing, in this context, becomes a process of restoring intuitive access through safe, attuned relationships and the gradual rebuilding of interoceptive capacity (van der Kolk, 2014; Porges, 2011).

Healing restores this connection on a cognitive, physiological, and relational level. Through attuned therapeutic relationships and practices that foster interoception, the nervous system learns to settle, recalibrate, and track subtle internal signals once again (Porges, 2011; Ogden et al., 2006; Siegel, 2012). As integration occurs, previously fragmented or overwhelming sensations are brought into conscious awareness. This shift reestablishes the body’s role as a site of truth and guidance, transforming it from a locus of unintegrated, conflicting information into an integrated compass for efficient decision making.

## Discussion

Throughout this investigation of the complex interplay between nature and nurture, it becomes apparent that intuitive attunement is not a gift born only to a select a few, but a biological potential present in all humans, although one that is more frequently cultivated, reinforced, and restored in women due to a convergence of evolutionary, hormonal, social, and experiential forces. Through pregnancy, caregiving, emotional socialization, and the often invisible labor of relational caretaking, women become finely attuned to their internal states and to the nervous systems of others.

By tracing intuition to its biological foundations, cultural reinforcements, and reparative pathways, this paper repositions attunement as a grounded and embodied form of intelligence that arises from the integration of embodied experience into a functioning and informed nervous system. Attunement, which has a foundational role in many if not most aspects of intuition, is the body’s way of knowing. It is the nervous system’s capacity to read the emotional weather, to co-regulate, to sense danger, care, or truth before words are even spoken.

As a human survival strategy, intuition is a relational skill, and a somatic inheritance, particularly honed, but not owned, by women. As we become an increasingly interconnected species and are faced with navigating complex sociocultural landscapes, it is clear that intuition becomes increasingly valuable. Women’s heightened intuitive capacities, developed through biologically influenced child rearing and caregiving roles and further enhanced through sociocultural dynamics, may play a pivotal role in facilitating comprehension and communication in a world in which individuals are increasingly traumatized and thus internally fragmented. Understanding intuition as a product of both nature and nurture allows for a more nuanced appreciation of its role in cognitive processes, decision-making, and social interactions. Moreover, the connection between trauma healing and intuition highlights how societal structures and therapeutic interventions can influence intuitive development. Encouraging emotional intelligence and interoceptive awareness across all genders may contribute to a more balanced approach to cognition and decision-making.

Yet, as we intimate above, this capacity is not without fragility. Trauma can sever the connection between sensation and meaning, disrupting the internal compass. But healing through co-regulation, somatic awareness, and relational safety offers a path back. In fact, post-traumatic growth can yield deeper intuitive clarity. What was once hypervigilance can become attunement; what was dissociation can become presence.

While our hypothesis is supported by various lines of evidence, we acknowledge the need for more research. Future studies might focus on developing more precise methods for measuring intuitive cognitive processes, examining how these abilities translate from caregiving to other domains, and exploring the potential for enhancing intuitive abilities in all individuals, independent of gender. In addition, future research should explore how these skills can be leveraged in fields such as leadership, healthcare, and conflict resolution.

Finally, we posit that it is the responsibility of all members of society–men and women alike–to develop this crucial and beneficial skill of intuition. We leave the reader with the open-ended inquiry of how we as a collective can design systems that better support both men and women in developing their intuitive skills and providing the nurturance and resources necessary for all humans to work on integrating body and mind and come back to a whole and aligned self. By recognizing the value of intuition, we as a collective can foster a more holistic approach to intelligence that integrates both analytical and embodied forms of knowledge.
